# Formation of Tumorspheres with Increased Stemness without External Mitogens in a Lung Cancer Model

**DOI:** 10.1155/2016/5603135

**Published:** 2016-01-06

**Authors:** Juan Sebastian Yakisich, Neelam Azad, Rajkumar Venkatadri, Yogesh Kulkarni, Clayton Wright, Vivek Kaushik, Anand Krishnan V. Iyer

**Affiliations:** Department of Pharmaceutical Sciences, School of Pharmacy, Hampton University, Hampton, VA 23668, USA

## Abstract

Like with most solid tumors, the presence of a subpopulation of cancer stem cells (CSCs) or cancer stem-like cells (CS-LCs) has been associated with chemoresistance and tumor relapse in lung cancer cells. In the absence of serum, CSCs/CS-LCs have the ability to grow as lung tumorspheres (LTSs), and this system is routinely used for isolation and characterization of putative CSCs/CS-LCs. Methods to isolate LTSs are usually performed in serum-free media supplemented with specific additives such as epidermal growth factor and basic fibroblast growth factor. In this study, we report the generation of LTSs without the addition of any external mitogenic stimulation. LTSs generated in this manner demonstrated several traits usually associated with increased stemness such as elevated expression of the stemness-associated marker Sox2 and increased chemoresistance to conventional anticancer drugs. In addition, we report that the FDA-approved drug Digitoxin, at concentration close to its therapeutic level, decreased the viability of LTSs and downregulated Sox2 independent of the PI3K/AKT pathway. The potential use of LTSs generated without the addition of any external mitogenic stimulation to study the role of specific factor(s) associated with stemness properties is also discussed.

## 1. Introduction

Lung cancer is a devastating disease with an overall five-year survival rate of only 16% [1]. It is the leading cause of cancer-related deaths among both men and women, with an estimated 228,190 reported new cases of lung cancer in the US and 159,480 deaths from the disease in 2013 [2].

Like with other cancers, the presence of a subpopulation of cancer stem cells (CSCs) or cancer stem-like cells (CS-LCs) has been associated in lung cancer with chemoresistance and tumor relapse. CSCs/CS-LCs were initially described as a rare subpopulation of cancer cells with unlimited self-renewal capacity and ability to differentiate and repopulate the entire tumor. The classical CSC model proposed a hierarchical organization where only CSCs can generate non-CSCs. However, emerging experimental evidence suggests that cancer cells are extremely plastic in terms of stemness, and non-CSCs can also generate CSCs [3–5]. For this reason, elucidating the underlying mechanism(s) responsible for the regulation of stemness as well as the identification of factors involved in its modulation is of importance in developing novel therapeutic modalities for cancer treatment.

Methods to isolate/enrich and characterize subpopulations of cancer cells with increased stemness properties are useful in cancer research. Putative CSCs/CS-LCs have been isolated by several methods based on either the expression of surface markers or intrinsic functional properties (side population, PKH26 and PKH6 retention) (reviewed by Tirino et al. [6]). The sphere-formation assay is another widely used method to isolate and characterize putative CSCs/SC-LCs, and it is thought that the ability to form clonal spheres is a unique characteristic of CSCs [6, 7]. Methods to isolate cancer stem-like cells as spheres (tumorspheres) are usually done in serum-free media containing exogenous mitogens such as epidermal growth factor (EGF), basic fibroblast growth factor (bFGF), or both [7, 8]. Additional supplements such as insulin, insulin-transferrin-sodium selenite (combination of insulin, transferrin, and selenite), B27, and hydrocortisone are also utilized [9–13]. Tumorspheres can induce an enrichment in CSC but only in a cell line-dependent manner, and tumorspheres-forming cells sometimes display lower tumorigenic potential than adherent cell [14]. CSCs/CS-LCs have been isolated from the NCI-H460 lung cancer cell line [10, 15, 16] and are associated with chemoresistance to anticancer drugs such as Cisplatin, Etoposide, Doxorubicin, and Paclitaxel [13, 15, 17, 18]. One hallmark of cancer cells in general is their ability to sustain proliferative signaling and divide in the absence of exogenous mitogenic stimulation, leading to unregulated proliferation [19]. This has been demonstrated for glioma CSCs/CS-LCs that can form spheres in serum-free media without exogenous mitogens [20, 21]. Serum-free media can successfully support the growth of CSCs, and short periods (48 h) of serum starvation were also shown to enrich the side population in three different cancer cell lines [22].

Cancer cells growing as spheres in the absence of exogenous mitogenic stimulation not only provide further support for the independence of external signal but can also provide an important experimental system to study the role of specific factors in the basal signaling network responsible for maintenance of the stemness status and the regulation of their key components.

In this study, we demonstrated the ability of NCI-H460 lung cancer cells to grow in the absence of external mitogens and evaluated several stemness properties such as the ability to form spheres, the expression of the stemness-associated protein Sox2, the expression of the epithelial-mesenchymal transition (EMT) associated marker Vimentin, and the resistance of LTSs to classical anticancer drugs.

## 2. Materials and Methods

### 2.1. Chemicals and Reagents

#### 2.1.1. Drugs

Hydroxyurea (HU), Paclitaxel (PX), Colchicine (CX), Digitoxin (DIG), Poly-HEMA (poly(2-hydroxyethyl methacrylate)), and MTT (Thiazolyl Blue Tetrazolium Bromide) were purchased from Sigma-Aldrich (St. Louis, MO). HU and CX were prepared as stock solution (500 mM and 10 mM, resp.) in distilled sterile water and stored in aliquots at −20°C. PX and DIG were prepared as stock solution (1 mM and 10 mM, resp.) in DMSO and stored in aliquots at −20°C.

### 2.2. Cell Culture

The human lung epithelial cancer cell line NCI-H460 was obtained from American Type Culture Collection (Manassas, VA). NCI-H460 cells were cultured in complete media (CM) = RPMI 1640 (Hyclone) supplemented with 5% FBS, 2 mM L-glutamine, 100 U/mL penicillin, and 100 mg/mL streptomycin. All cells were cultured in a 5% CO_2_ environment at 37°C.

### 2.3. Generation of Lung Tumorspheres (LTSs)

For spheres isolation, cells grown in CM (70–80% confluency) were cultured overnight in serum-free media (SFM, same as CM but without FBS). Then, cells were trypsinized and incubated in SFM for 7–45 days in Poly-HEMA coated plate to prevent attachment. LTSs were observed as early as 72 h after plating. For maintenance of LTSs, one-third of the SFM was replaced every 3-4 days. LTSs grown in SFM for >14 days were used for subsequent experiments.

### 2.4. Short-Term Antiproliferative Assay (MTT Assay and CCK Assay)

For adherent cultures (parental H460 cells), cells were plated in 96-well cell-culture microplates (Costar, USA) at ~2,000 cells per well and incubated overnight in CM. Then, the cells were exposed to the appropriate concentration of drug or vehicle for 72 h. Cell viability for adherent cells was evaluated by the MTT assay. The absorbance of solubilized formazan was read at 570 nm using Gen 5 2.0 All-In-One microplate reader (BioTek Instruments, Inc.).

For floating LTSs, cells growing in Poly-HEMA plates were collected in 15 mL Falcon tubes, centrifuged at 700 rpm × 3 min, and resuspended in fresh SFM. In order to plate the same number of cells, this cell suspension was split in 1 mL aliquots. Vehicle or drugs were added to each aliquot and then 150 *μ*L cell suspension was loaded into each microwell (in a 96-well plate) and incubated for 72 h. For floating LTSs, cell viability was evaluated by the CCK-8 assay (Dojingo Laboratories).

In all cases, the highest concentration of DMSO was used in the control and this concentration was maintained below 0.001% (v/v). This DMSO concentration did not show any significant antiproliferative effect on the cell lines or LTSs in a short-term assay.

### 2.5. Western Blotting

Preparation of cell lysates and western blotting were performed as described previously [23]. Antibodies for Sox2, Bcl2, Vimentin, AKT, pAKT, *β*-actin, and peroxidase-conjugated secondary antibody were purchased from Cell Signaling Technology (Danvers, MA). Antibody for GAPDH was purchased from Santa Cruz Biotechnology (Dallas, TX). The blotting membranes were probed with 1 : 1000 diluted antibody for Sox2, Vimentin, Bcl2, *β*-actin, and GAPDH and 1 : 4000 for the peroxidase-conjugated secondary antibody. Immune complexes were detected by chemiluminescence using SuperSignal West Femto Maximum Sensitivity Substrate (Thermo Fisher Scientific, Grand Island, NY) and photographed using myECL imager instrument (Thermo Fisher Scientific, Grand Island, NY).

### 2.6. Statistical Analysis

All Pairwise Multiple Comparison Procedures (ANOVA, Student-Newman-Keuls Method) in Figure 3 have been done using SigmaPlot (V. 11.0) software.

## 3. Results

### 3.1. NCI-H460 Cells Have the Ability to Grow as Floating Lung Tumorspheres without Exogenous Mitogenic Stimulation

**Figure 1:**
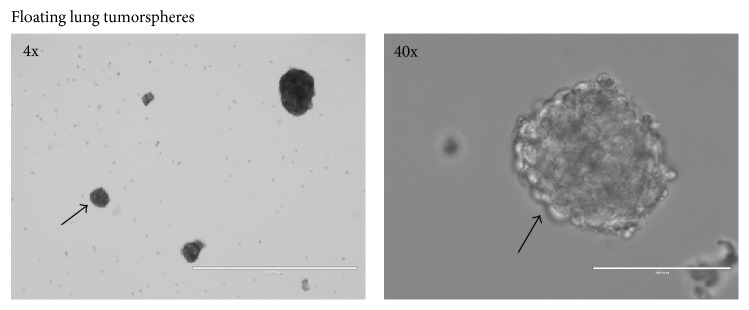
Morphology of H460 cells grown as spheres without addition of external mitogens. Bars are 1000 *μ*m (4x) and 100 *μ*m (40x). The arrow indicates the same sphere at two different magnifications.

We first investigated the ability of H460 cells to grow as spheres in SFM without the addition of any mitogenic factors. Adherent H460 cells growing under routine culture conditions (RCCs) in complete media were incubated overnight in SFM. Then, these cells were trypsinized and cultured in SFM in Poly-HEMA coated plates. Under these conditions, H460 cells spontaneously formed spheres as early as within 72 h which could be propagated and maintained for several months.  Figure 1 shows representative images of LTSs.

### 3.2. Lung Tumorspheres Express Higher Levels of the Stemness-Associated Protein Sox2

**Figure 2:**
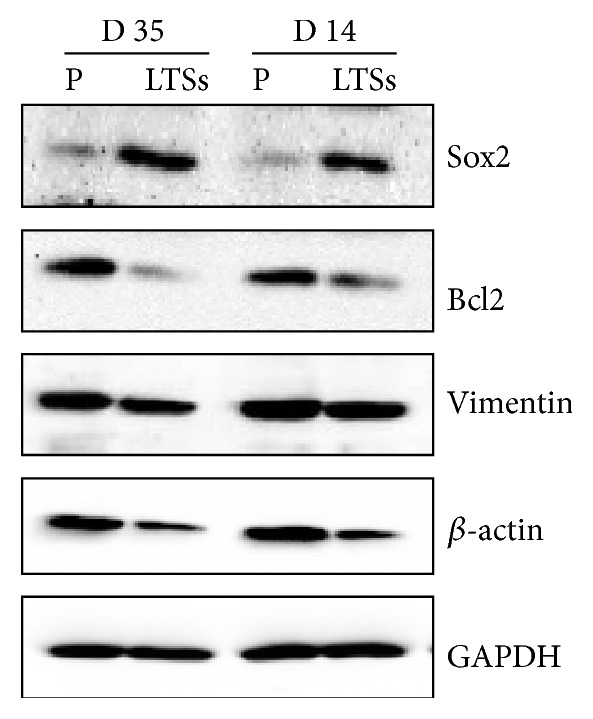
Expression of Sox2, Bcl2, Vimentin, *β*-actin, and GAPDH in H460 parental cells (P) and lung tumorspheres (LTSs) grown in SFM without external mitogens for 14 and 35 days.

We next evaluated the expression of Sox2 by western blot. Sox2 expression was high in LTSs compared to the parental H460 cell line.  Figure 2 shows that the expression of Sox2 in LTSs grown in SFM for 14 and 35 days is higher compared to the parental cell line H460.

### 3.3. Lung Tumorspheres Express Lower Levels of Vimentin, Bcl2, and *β*-Actin

We also evaluated the expression of Vimentin, a protein associated with the epithelial-mesenchymal transition, Bcl2, a key regulator of apoptosis, and *β*-actin, a protein used commonly as control for loading. All these proteins showed decreased expression levels in LTSs compared to the parental cell line H460 (Figure 2). GAPDH was used as loading control.

### 3.4. H460 Lung Tumorspheres Are Resistant to Conventional Anticancer Drugs

**Figure 3:**
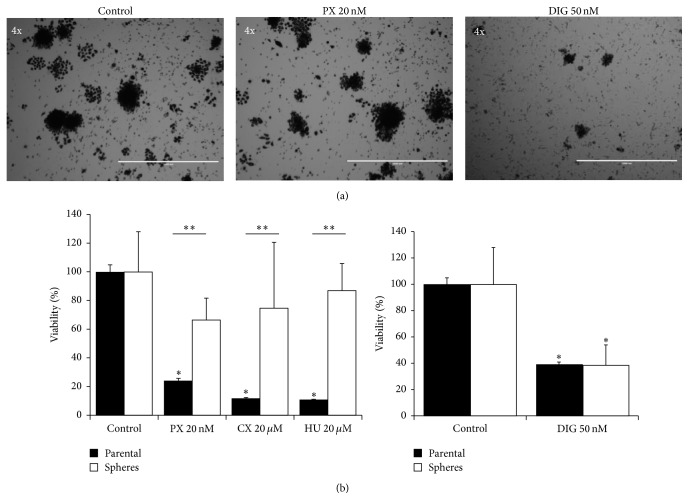
Chemosensitivity of lung tumorspheres compared to the parental H460 cell line. (a) Representative images of lung tumorspheres treated with DMSO (control), Paclitaxel (PX), or Digitoxin (DIG). In these images, the dark appearance of the tumorspheres is because pictures have been taken after incubation with MTT solution to show that the cells are metabolically active and able to reduce the MTT. Bars are 1000 *μ*m (4x). (b) Quantification of the effects of different anticancer agents on parental H460 cells (black columns) and LTSs (white columns). In all cases, H460 parental cells and lung tumorspheres were incubated with the indicated concentration of drugs for 72 h. Cell viability was assessed by the MTT assay for H460 parental cells and by the CCK assay for lung tumorspheres. Data (mean ± ES) are representative of three independent experiments performed in sextuplicate. ^*∗*^ *P* < 0.05 compared to control value. ^*∗∗*^ *P* < 0.05 compared between parental and spheres.

Subpopulations of H460 cells growing under specific culture conditions (parental cells and LTSs) were used for testing their sensitivity to three different drugs. The concentration of each tested drug was greater than its IC_50_ as determined previously [24] by the MTT assay (72 h, in CM): PX 20 nM, CX 20 *μ*M, and HU 2 mM. While the parental H460 cell line when growing under routine culture conditions as adherent cell was highly sensitive to PX, HU, and CX, these drugs did not significantly affect the viability of LTSs (Figures 3(a) and 3(b), top panel).

### 3.5. H460 Lung Tumorspheres Are Sensitive to Digitoxin (DIG)

We also tested the effect of DIG on the viability of lung tumorspheres. We used a fixed concentration of 50 nM based on previous concentration-dependent experiments performed in the parental H460 cell line where IC_50_ was ~20 nM [24]. DIG decreased the viability of LTSs by approximately 60% (Figure 3(b), right panel).

### 3.6. DIG Downregulates Sox2 Expression in LTSs Likely Independent of the PI3K/AKT Pathway

**Figure 4:**
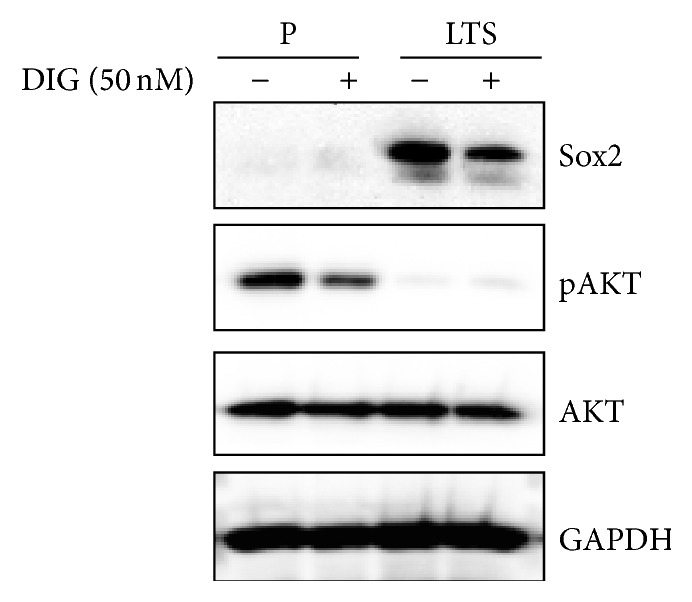
DIG downregulates Sox2 expression likely independent of the PI3K/AKT pathway. Expression of Sox2, pAKT, AKT, and GAPDH in untreated and DIG-treated H460 parental cells (P) and lung tumorspheres (LTSs) grown in SFM without external mitogens for 14 days.

Since PI3K/AKT-mediated Sox2 expression has been associated with resistance to conventional anticancer drugs, we measured the expression of Sox2, AKT, and pAKT. LTSs and parental H460 cells were treated with DMSO alone (control) or with DIG (50 nM) for 72 h. Consistent with our results shown in Figure 2, the basal Sox2 expression was very low in parental H460 cells compared to LTSs. DIG-treated LTSs showed lower levels of Sox2 compared to DMSO treated cells (Figure 4). On the contrary, while the basal levels of total AKT expression showed similar levels in both parental H460 and LTSs cells, pAKT expression was very low in LTSs compared to parental cells. Treatment with DIG 50 nM did not affect the levels of total AKT but decreased pAKT expression in parental H460 cells but not in LTSs. We also assessed the expression of Nanog and Wnt5a/b, both proteins that have been identified as stem cell markers [9, 25, 26]. Our result indicated that while LTSs and parental H460 cells express similar basal levels of Nanog, the basal levels of Wnt5a/b were significantly higher in LTSs (data not shown).

## 4. Discussion

Lung cancer tumorspheres are typically isolated in serum-free media supplemented with external mitogens such as epidermal growth factor (EGF), basic fibroblast growth factor (bFGF), and insulin [10, 15, 16]. In this study, we demonstrated that H460 cells can grow without the addition of external mitogen and that they are able to generate spheres (tumorspheres) when cultured in Poly-HEMA coated plates (Figure 1). Such ability to form tumorspheres without mitogenic supplementation was demonstrated only for glioblastoma cells [21]. LTSs were observed after 3-4 days after plating in Poly-HEMA coated plates which is consistent with previous reports where the side population of H460 cells was able to generate LTSs within 4 days in the presence of mitogens [16]. LTSs grown without exogenic mitogenic stimulation also showed increased levels of the stemness-associated protein Sox2 (Figure 2). Sox2 is a stem cell transcription factor involved in maintenance of stemness related processes such as clonogenicity, pluripotency, and self-renewal [27–29]. The Sox2 gene is frequently amplified in small-cell lung cancer cells obtained from primary cells and cell lines [30].

We also evaluated the expression of Vimentin and Bcl2 that are expected to be elevated in CSCs. However, the levels of these two proteins were lower in LTSs compared to the parental cell line. Vimentin is a mesenchymal gene usually upregulated during epithelial-mesenchymal transition (EMT), a process associated with cancer CSCs/CS-LCs. On the other hand, increased levels of Bcl2, a prosurvival protein, are associated with chemoresistance [31]. Despite loading equal amount of protein in western blot experiments, the levels of *β*-actin, a housekeeping protein widely used as a loading control, were consistently lower in LTSs compared to the parental cell line. For this reason, we also used GAPDH as an additional loading control that showed similar level in both LTSs and the parental cell line (Figure 2). One possible explanation for these results that may require further evaluation is that, in our LTSs formed in the absence of external mitogenic factors, the activity of signaling pathways that stimulate the expression of these proteins is lower than control. For instance, bFGF that is routinely included in media to isolate putative CSCs/CS-LSCs is a pleiotropic growth factor that* per se* increases the stemness of human stem cells from the apical papilla [32]. Therefore, the presence of bFGF during the preparation of LTSs may potentially increase the expression of proteins that otherwise will not be expressed when spheres are generated in its absence, generating artefactual responses due to the mitogenic factor itself. Since growth factors can affect multiple signaling pathways and cellular functions, the method described here for the generation of LTSs in the absence of external mitogens could be useful to investigate the role of one or more specific factors on the stemness properties of cancer cells. Extensive rearrangement of the cytoskeleton may also explain the lower levels of *β*-actin found in LTSs compared to the parental H460 cell line.

The LTSs growing in the absence of external mitogenic factors showed elevated resistance to conventional anticancer drugs such as PX, CX, and HU (Figure 3) which is a trait usually found in CSCs/CS-LCs [6]. Lung CSCs are known to be resistant to PX [17] and other conventional anticancer drugs such as Cisplatin, Doxorubicin, and Etoposide [15].

We recently reported that DIG, an FDA-approved drug for treatment of cardiac disease, has potent antiproliferative effects against H460 cells growing both under routine culture conditions and under prolonged (7–10 days) serum starvation [24]. Since CSCs/CS-LCs typically grow without serum, we investigated the effect of DIG on LTSs growing without any external mitogenic stimulation. Figure 3 shows that DIG at 50 nM decreased the viability of lung tumorspheres by approximately about 60%. This is of clinical importance since the therapeutic plasma levels of DIG are considered to be in the ranges of 13 to 33 nM [33, 34] and up to 46 nM [35]. Sox2, via the PI3K/AKT pathway, has been recently shown to be involved in resistance to conventional anticancer drugs such as Paclitaxel in prostate [36] and ovarian [37] cancers. The PI3K/AKT pathway is activated by external mitogens upon binding to receptor tyrosine kinases (RTK) such as EGFR, HER2, and IGF [38]. We found that the basal levels of pAKT were extremely low in LTSs compared to parental H460 cells despite having similar basal levels of total AKT. DIG decreased the levels of pAKT in parental H460 cells but did not have any effects in LTSs (Figure 4). This finding indicates that the PI3K/AKT pathway is largely inactive in LTSs, probably due to lack of external mitogenic stimulation, suggesting that DIG exerts its antiproliferative effects at least in part by downregulating Sox2 but not via the PI3K/AKT pathway. Overall, our data suggests a potential role for DIG and other cardiac glycosides in the treatment of cancers that are highly resistant to traditional chemotherapeutic regimens.

## 5. Conclusion

In summary, we report for the first time that H460 lung cancer cells can grow as tumorspheres in the absence of any external mitogenic factor. These spheres show increased stemness properties such as resistance to conventional anticancer drugs and overexpression of the stemness-associated marker Sox2. In addition, we showed that these cells are sensitive to the clinically used drug DIG at a concentration close to the therapeutic level and that this effect is at least partially mediated by downregulation of Sox2 independently of the PI3K/AKT pathway.
